# A 15-year consolidated overview of data in over 6000 patients from the Transthyretin Amyloidosis Outcomes Survey (THAOS)

**DOI:** 10.1186/s13023-023-02962-5

**Published:** 2023-11-10

**Authors:** Luca Gentile, Teresa Coelho, Angela Dispenzieri, Isabel Conceição, Márcia Waddington-Cruz, Arnt Kristen, Jonas Wixner, Igor Diemberger, Juan Gonzalez-Moreno, Eve Cariou, Mathew S. Maurer, Violaine Planté-Bordeneuve, Pablo Garcia-Pavia, Ivailo Tournev, Jose Gonzalez-Costello, Alejandra Gonzalez Duarte, Martha Grogan, Anna Mazzeo, Doug Chapman, Pritam Gupta, Oliver Glass, Leslie Amass, the THAOS investigators

**Affiliations:** 1 University of Messina, Messina, Italy; 2 Unidade Corino Andrade, Centro Hospitalar Universitário de Santo António, Porto, Portugal; 3 Division of Hematology, Mayo Clinic, Rochester, MN USA; 4 CHULN- Hospital de Santa Maria, FML, Universidade de Lisboa, Lisbon, Portugal; 5 Federal University of Rio de Janeiro, National Amyloidosis Referral Center, CEPARM, Rio de Janeiro, Brazil; 6 Department of Cardiology, Angiology, Respiratory Medicine, Medical University of Heidelberg, Heidelberg, Germany; 7 Department of Public Health and Clinical Medicine, Umeå University, Umeå, Sweden; 8 Department of Medical and Surgical Sciences, DIMEC, University of Bologna, Bologna, Italy; 9 Cardiology Unit, IRCCS Policlinico di S. Orsola, Bologna, Italy; 10 Hospital Son Llatzer, Palma de Mallorca, Spain; 11 Department of Cardiology, University Hospital Rangueil, Toulouse, France; 12 Columbia University College of Physicians and Surgeons, New York, NY USA; 13 Hopital Henri Mondor, East Paris-Créteil University, Assistance Publique-Hopitaux de Paris, Créteil, France; 14 Hospital Universitario Puerta de Hierro Majadahonda, CIBERCV, Madrid, Spain; 15 Centro Nacional de Investigaciones Cardiovasculares, Madrid, Spain; 16 Clinic of Nervous Diseases, Department of Neurology, UMBAL Aleksandrovska, Medical University-Sofia, Sofia, Bulgaria; 17 Department of Cognitive Science, New Bulgarian University, Sofia, Bulgaria; 18 Hospital Universitari de Bellvitge, IDIBELL, CIBER-CV, Barcelona, Spain; 19 NYU Langone School of Medicine, New York, NY USA; 20 Instituto Nacional de Ciencias Médicas y Nutrición Salvador Zubirán, Mexico City, Mexico; 21 Department of Cardiovascular Diseases, Mayo Clinic, Rochester, MN USA; 22 Pfizer Inc, New York, NY USA; 23 Pfizer Healthcare India Pvt Ltd, Chennai, India

**Keywords:** Amyloidosis, Cardiomyopathy, Polyneuropathy, Transthyretin, Registry

## Abstract

**Background:**

Transthyretin amyloidosis (ATTR amyloidosis) is a progressive, multisystemic, life-threatening disease resulting from the deposition of variant or wild-type (ATTRwt amyloidosis) transthyretin amyloid fibrils in various tissues and organs.

**Methods:**

Established in 2007, the Transthyretin Amyloidosis Outcomes Survey (THAOS) is the largest ongoing, global, longitudinal, observational study of patients with ATTR amyloidosis, including both hereditary and wild-type disease, and asymptomatic carriers of pathogenic *TTR* mutations. This analysis describes the baseline characteristics of symptomatic patients and asymptomatic gene carriers enrolled in THAOS since its inception in 2007 (data cutoff: August 1, 2022), providing a consolidated overview of 15-year data from the THAOS registry.

**Results:**

This analysis included 4428 symptomatic patients and 1707 asymptomatic gene carriers. The majority of symptomatic patients were male (70.8%) with a mean (standard deviation [SD]) age at symptom onset of 56.6 (17.9) years. Compared with the 14-year analysis, V30M remained the most prevalent genotype in Europe (62.2%), South America (78.6%), and Japan (74.2%) and ATTRwt remained most common in North America (56.2%). Relative to the 14-year analysis, there was an increase of mixed phenotype (from 16.6 to 24.5%) and a reduction of predominantly cardiac phenotype (from 40.7 to 31.9%). The proportion of patients with predominantly neurologic phenotype remained stable (from 40.1 to 38.7%). Asymptomatic gene carriers were 58.5% female with a mean age at enrollment of 41.9 years (SD 15.5).

**Conclusions:**

This overview of > 6000 patients enrolled over 15 years in THAOS represents the largest registry analysis of ATTR amyloidosis to date and continues to emphasize the genotypic and phenotypic heterogeneity of the disease. Nearly a quarter of the symptomatic population within THAOS was mixed phenotype, underscoring the need for multidisciplinary management of ATTR amyloidosis.

***Trial registration*:**

*ClinicalTrials.gov Identifier*: NCT00628745.

**Supplementary Information:**

The online version contains supplementary material available at 10.1186/s13023-023-02962-5.

## Introduction

Transthyretin amyloidosis (ATTR amyloidosis) is a progressive, multisystemic, life-threatening disease characterized by deposits of amyloid fibrils in the peripheral nerves, heart, and other tissues and organs, resulting in polyneuropathy, cardiomyopathy, or a mix of both neurologic and cardiac manifestations [1–3]. ATTR amyloidosis may be caused by one of over 130 pathogenic mutations that destabilize the TTR protein (hereditary ATTR amyloidosis [ATTRv amyloidosis]) or the accumulation of non-mutated TTR protein (wild-type ATTR amyloidosis [ATTRwt amyloidosis]) [3, 4]. The phenotypic presentation of ATTRv amyloidosis is clinically heterogeneous and can be predominantly neurologic, predominantly cardiac, or mixed phenotype, depending on the particular *TTR* variant and other factors [2, 5]. ATTRwt amyloidosis most often presents as cardiomyopathy [1]. If left untreated, median survival estimates in patients with ATTR amyloidosis range from 2 to 10 years, depending on genotype and other factors [1, 2, 6]. Diagnosing ATTR amyloidosis can be difficult due to low disease awareness, indeterminate family history, and the heterogeneity of clinical presentation that can overlap with more common diseases [2, 6, 7]. Better clinical characterization of the disease may lead to earlier identification and intervention, which is associated with improved outcomes [8].

Established in 2007, the Transthyretin Amyloidosis Outcomes Survey (THAOS) is an ongoing, global, longitudinal, observational survey of patients with ATTR amyloidosis, including both hereditary and wild-type disease, and asymptomatic carriers with *TTR* mutations [9]. THAOS collects multinational, longitudinal data on the natural history of the disease from a large, diverse patient population to help inform the characterization of ATTR amyloidosis and improve disease diagnosis and patient management. This analysis provides an annual update [10] of the characteristics of patients with ATTR amyloidosis (both ATTRv and ATTRwt) and asymptomatic gene carriers at the time of their enrollment into THAOS, providing a global overview of THAOS data since its inception 15 years ago.

## Methods

### Study design and patient population

The study design and eligibility criteria of THAOS have been described [11]. All study sites received ethical or institutional review board approval prior to patient enrollment, and all patients provided written informed consent. The Good Pharmacoepidemiology Practice guidelines and the principles of the Declaration of Helsinki were duly followed for this study.

The analysis population comprised all patients enrolled in THAOS (data cutoff date: August 1, 2022). The definitions of symptomatic status, asymptomatic status, and those with missing symptomatic status have been described previously [10]. Demographics, clinical characteristics, and patient-reported outcomes collected at enrollment were analyzed in the overall cohort of symptomatic patients and by the following genotype subgroups: ATTRwt amyloidosis, V30M (p.V50M) with early-onset disease (age ≤ 50 years, based on age at diagnosis), V30M with late-onset disease (age > 50 years), and non-V30M.

Phenotype at enrollment was analyzed in symptomatic patients by region as previously described [10]. Patients were classified by phenotype based on the following definitions: Predominantly cardiac phenotype included patients with abnormal electrocardiogram (ECG) due to rhythm disturbance, heart failure, or dyspnea and no more than mild neurologic or gastrointestinal (GI) symptoms (excluding erectile dysfunction, constipation, and carpal tunnel); cardiac symptoms did not need to be ongoing at a given visit to be included for phenotyping, but symptoms had to be definitely ATTR amyloidosis related. Predominantly neurologic phenotype included patients with neurologic or GI symptoms of any severity and without abnormal ECG due to rhythm disturbance, heart failure, or dyspnea; neurologic and GI symptoms had to be ongoing and definitely ATTR amyloidosis related. A modified Polyneuropathy Disability (mPND) score ≥ I was considered a neurologic symptom in this analysis, whereas it was not in prior analyses [10]. Mixed phenotype included patients with abnormal ECG due to rhythm disturbance, heart failure, or dyspnea, and neurologic or GI symptoms of any severity, but who did not satisfy criteria for a predominantly cardiac or predominantly neurologic phenotype. Unknown phenotype included all other symptomatic patients who did not meet any of the above criteria for either predominantly cardiac, predominantly neurologic, or mixed phenotypes. All patients with ATTRwt amyloidosis were classified as predominantly cardiac at enrollment unless they had any neurologic symptoms definitely related to ATTR amyloidosis, in which case they were classified as having a mixed phenotype.

The categorization of symptoms and their manifestations have been described [10]. Demographics collected at enrollment were also summarized for the asymptomatic carriers overall and by genotype category (V30M and non-V30M).

### Assessments

Assessments have been described in detail [10]. Briefly, a patient’s ability to perform normal life activities and need for assistance was assessed in symptomatic patients using the Karnofsky Performance Status Scale Score. Neurologic impairment was measured in symptomatic patients using the derived Neuropathy Impairment Score in the Lower Limbs (NIS-LL) and mPND scores. Cardiac measures included select echocardiographic measures, N-terminal pro-B-type natriuretic peptide (NT-proBNP) concentration, and New York Heart Association (NYHA) class. Quality of Life (QoL) was assessed using the EQ-5D-3L and the Norfolk Quality of Life – Diabetic Neuropathy questionnaire.

### Statistical analyses

This was a descriptive analysis. Continuous data are presented as mean (standard deviation [SD]) or median (10th, 90th percentile), and categorical data are presented as count (percentage).

## Results

### Demographics and genotype

**Fig. 1:**
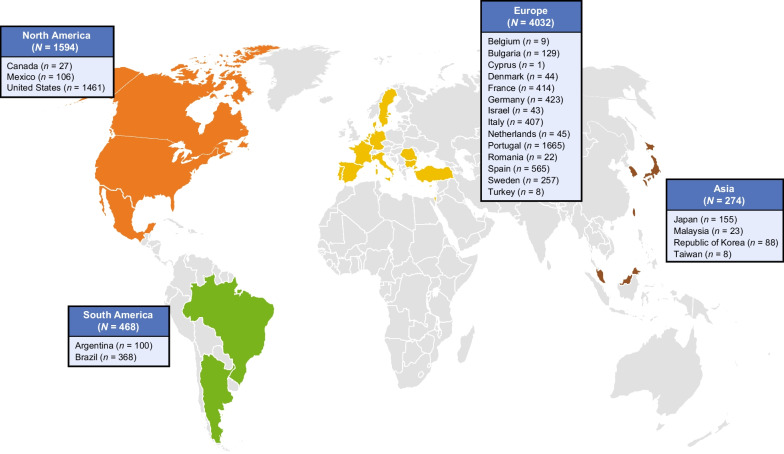
Geographic distribution of all patients enrolled in the Transthyretin Amyloidosis Outcomes Survey (THAOS)

**Fig. 2:**
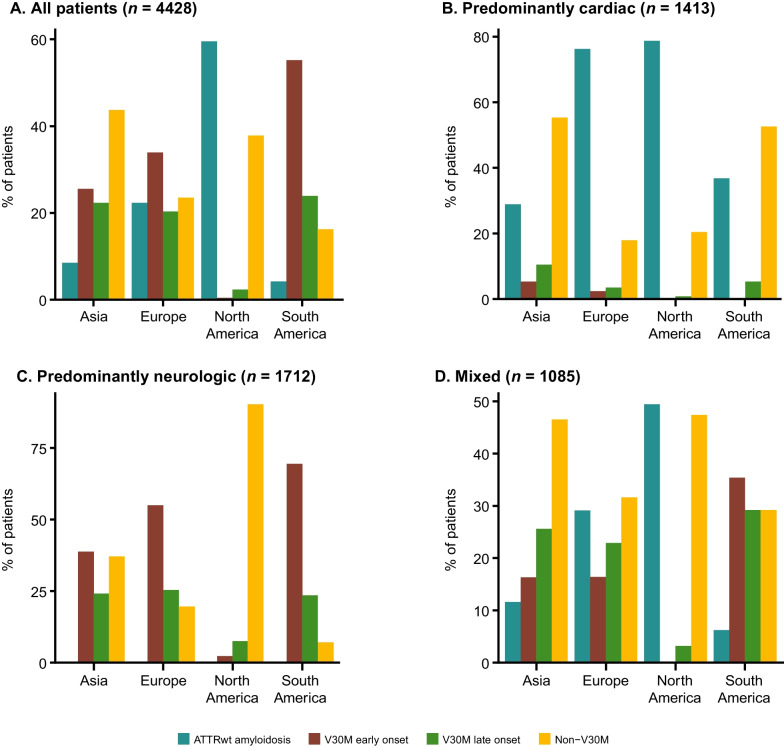
Regional distribution of genotype subgroups in symptomatic patients. The proportion of patients with each genotype shown by region in **a** the overall population of symptomatic patients and in patients with **b** predominantly cardiac, **c** predominantly neurologic, and **d** mixed phenotypes. ATTRwt amyloidosis = wild-type transthyretin amyloidosis

There were 6368 patients from 85 study sites and 23 countries enrolled in THAOS at the data cutoff date (Fig. 1). This included 4428 symptomatic patients and 1707 asymptomatic gene carriers. There were 233 wild-type patients who, despite having all symptoms assessed, did not meet the definition for the symptomatic set [10]. V30M remained the most prevalent genotype enrolled in THAOS patients (48.0%) as was reported in the previous year’s report [10], followed by ATTRwt amyloidosis (25.2%) and V122l (p.V142I) (6.0%) (Additional file 1: Table S1). Among the symptomatic patients, V30M (early or late onset) was most commonly enrolled in Europe (54.2%), South America (79.5%), and Japan (75.4%), and wild-type disease was most commonly enrolled in North America (59.5%) (Fig. 2a; Additional file 2: Table S2). The non-V30M variant was more common (even more prevalent than the wild type) in some individual countries (Mexico, Bulgaria, Denmark, Israel, Italy, Romania, Turkey, Malaysia and Taiwan), although the overall trend showed that V30M was the predominant genotype. Among the symptomatic patients with a predominantly cardiac phenotype, the non-Val30Met subgroup accounted for more than half the patients in Asia (55.3%) and South America (52.6%) (Fig. 2b). The non-Val30Met subgroup also accounted for 90.2% of the patients with a predominantly neurologic phenotype in North America, whereas, for other regions (South America, Europe and Asia), the V30M genotype (early or late onset) was more prevalent (Fig. 2c). The ATTRwt genotype accounted for almost half (49.4%) of the symptomatic patients with a mixed phenotype in North America (Fig. 2d).

Symptomatic patients were predominantly male across all genotype subgroups (Table 1). Overall, the mean age at symptom onset was 56.6 years and was higher in patients with ATTRwt amyloidosis compared with the other genotype subgroups. Mean time from symptom onset to diagnosis was 4.0 years in all symptomatic patients and ranged from 2.8 years in the early-onset V30M subgroup to 4.6 years in the ATTRwt amyloidosis subgroup. V30M remained the most prevalent genotype among the symptomatic patients in South America (79.5%), Europe (54.2%), and Asia (including Japan; 47.8%). The majority of these patients had early-onset disease in each of these regions (Fig. 2a; Additional file 2: Table S2).

**Table 1:** Demographics of symptomatic patients according to genotype category

	Overall (*n* = 4428)	ATTRwt amyloidosis (*n* = 1410)	V30M early onset (*n* = 1082)	V30M late onset (*n* = 670)	Non-V30M (*n* = 1264)
Male, *n* (%)	3137 (70.8)	1315 (93.3)	565 (52.2)	433 (64.6)	822 (65.0)
Race/ethnicity^a^, *n* (%)					
White	2450 (77.2)	1141 (94.1)	263 (68.3)	382 (83.2)	663 (59.5)
African descent	310 (9.8)	38 (3.1)	33 (8.6)	18 (3.9)	221 (19.8)
American Hispanic	17 (0.5)	1 (0.1)	10 (2.6)	1 (0.2)	5 (0.4)
Latino American	136 (4.3)	8 (0.7)	22 (5.7)	4 (0.9)	102 (9.1)
Asian	245 (7.7)	18 (1.5)	56 (14.5)	53 (11.5)	118 (10.6)
Other	14 (0.4)	6 (0.5)	1 (0.3)	1 (0.2)	6 (0.5)
Age at enrollment (years), mean (SD)	62.5 (17.22)	77.9 (7.14)	40.6 (9.44)	68.6 (7.94)	60.9 (13.22)
Age at onset of ATTR amyloidosis symptoms (years)	*n* = 4421	*n* = 1409	*n* = 1082	*n* = 670	*n* = 1259
Mean (SD)	56.6 (17.93)	72.3 (9.73)	33.8 (7.19)	63.3 (8.14)	55.0 (13.88)
Time from symptom onset to diagnosis (years)	*n* = 4069	*n* = 1337	*n* = 993	*n* = 596	*n* = 1142
Mean (SD)	4.0 (5.96)	4.6 (6.73)	2.8 (4.74)	3.7 (3.96)	4.4 (6.61)
Follow-up time^b^ (years), mean (SD)	3.9 (3.20)	2.3 (1.94)	6.8 (3.29)	4.1 (3.03)	3.2 (2.61)

V30M early onset and late onset *n* based on all patients with available data for disease diagnosis

Symptom onset was the date of first occurrence of symptom(s) reported as definitely related to ATTR amyloidosis

*ATTR amyloidosis * transthyretin amyloidosis, *ATTRwt amyloidosis* wild-type transthyretin amyloidosis, *SD*  standard deviation

^a^ Denominator for race/ethnicity is the total of non-missing records

^b^ Follow-up time is based on all patients, from enrollment to last observation

In contrast to symptomatic patients, the asymptomatic carriers had a higher proportion of females overall (58.5%), as did the V30M subgroup (61.4%) (Table 2). The mean age at enrollment was 41.9 years overall and was higher in the non-V30M subgroup compared with V30M asymptomatic gene carriers.

**Table 2:** Demographics of asymptomatic gene carriers according to genotype category

	Overall (*n* = 1707)	V30M (*n* = 1272)	Non-V30M (*n* = 435)
Male, *n* (%)	708 (41.5)	491 (38.6)	217 (49.9)
Race/ethnicity^a^, *n* (%)			
Caucasian	670 (80.2)	407 (88.3)	263 (70.3)
African descent	86 (10.3)	29 (6.3)	57 (15.2)
American Hispanic	9 (1.1)	5 (1.1)	4 (1.1)
Latino American	38 (4.6)	10 (2.2)	28 (7.5)
Asian	28 (3.4)	7 (1.5)	21 (5.6)
Other	4 (0.5)	3 (0.7)	1 (0.3)
Age at enrollment (years), mean (SD)	41.9 (15.51)	39.4 (14.57)	49.3 (15.81)

*SD * standard deviation

^a^ Denominator for race/ethnicity is the total of non-missing records

### Distribution of phenotypes at enrollment in symptomatic patients

**Fig. 3:**
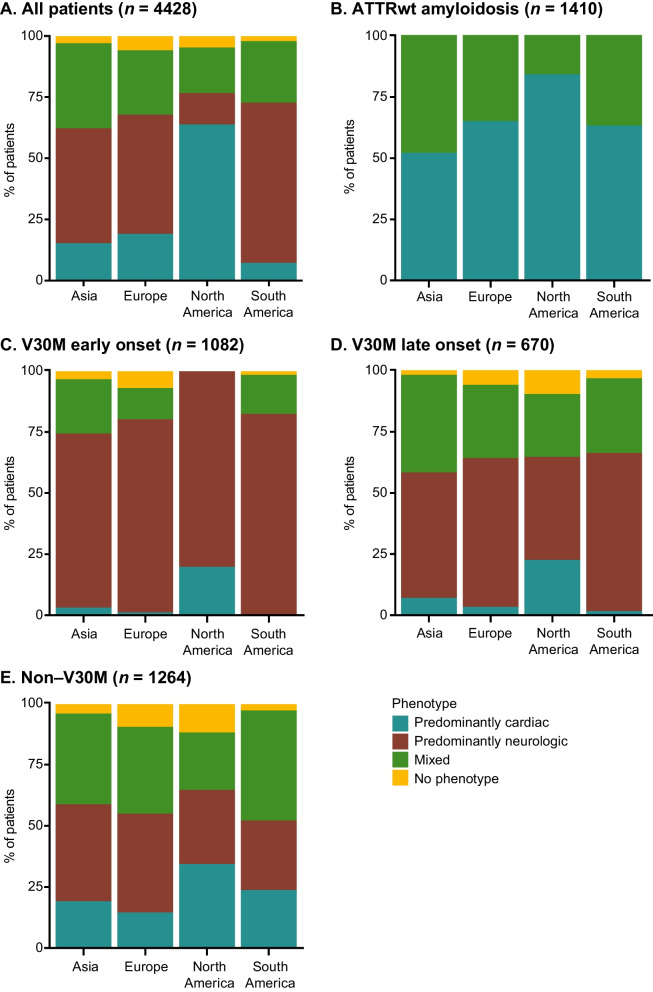
Regional distribution of phenotype at enrollment in symptomatic patients. The proportion of patients with each phenotype shown by region in **a** the overall population of symptomatic patients and by genotype category, **b** ATTRwt amyloidosis, **c** V30M early onset, **d** V30M late onset, **e** non-V30M. ATTRwt amyloidosis = wild-type transthyretin amyloidosis

The overall phenotype distribution for symptomatic patients at enrollment showed, in respect to last-year’s analysis, an increase in the proportion of mixed phenotype from 16.6% [10] to 24.5%. The predominantly cardiac (31.9%), predominantly neurologic (38.7%), and unknown (4.9%) phenotypes accounted for the remaining symptomatic patients (Additional file 2: Table S2). In North America, the majority of symptomatic patients had a predominantly cardiac phenotype overall (63.9%) as well as in the ATTRwt amyloidosis (84.5%) and non-V30M (34.4.%) subgroups (Fig. 3a; Additional file 2: Table S2). In South America, predominantly neurologic was the most prevalent phenotype at enrollment overall (65.6%) as well as in the V30M (early and late onset) subgroup (76.7%). The proportion of symptomatic patients with predominantly neurologic phenotype in Europe was 48.7% (Fig. 3a; Additional file 2: Table S2). In symptomatic patients with ATTRwt amyloidosis, 84.5% had a predominantly cardiac phenotype in North America, which was higher than in the other regions (Europe, South America and Asia; Fig. 3b). In Europe, the predominantly neurologic phenotype was found in 72.2% of patients in the V30M (early or late onset) subgroup, and in 40.6% of patients in the non-V30M subgroup (Fig. 3c, d, e; Additional file 2: Table S2). The mixed phenotype was more prevalent in Asian countries (excluding Japan) with an overall 40.6% of symptomatic patients (Additional file 2: Table S2).

The V30M late-onset subgroup had a greater proportion of mixed phenotypes versus the early-onset subgroup (30.6% vs. 13.8%) (Additional file 2: Table S2). The V30M late-onset predominantly cardiac phenotype showed a decrease in proportion among the symptomatic patients as compared to last year (4.3% vs. 1.4% [10]). The proportion of symptomatic patients with ATTRwt amyloidosis with a mixed phenotype at enrollment also showed a jump from 9.8% as observed in the previous year [10] to 23.9% (Additional file 2: table S2), but as discussed further below this result is due to a change in the definition of mixed phenotype in THAOS.

**Fig. 4:**
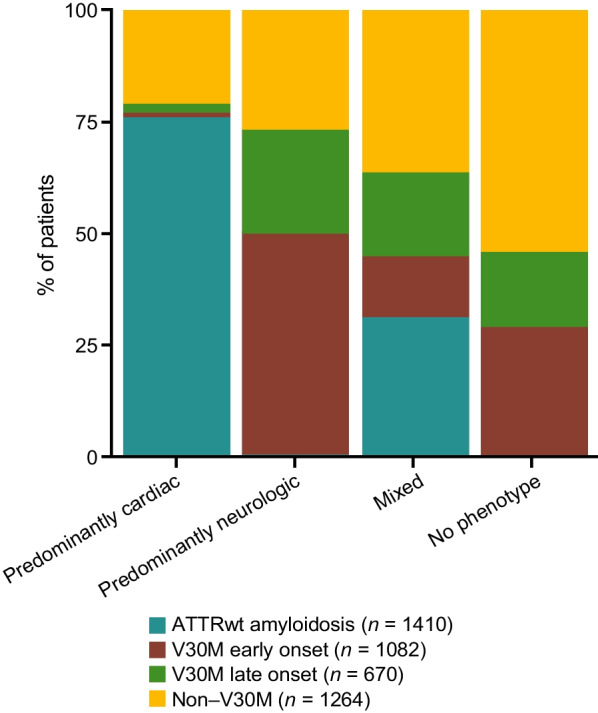
Distribution of phenotype at enrollment in symptomatic patients according to genotype category. The proportions of patients with each phenotype are shown by genotype. ATTRwt amyloidosis = wild-type transthyretin amyloidosis

The non-Val30Met subgroup was prevalent among symptomatic patients with a mixed phenotype (36.3%; Fig. 4). The V30M early onset group accounted for half of the symptomatic patients with a predominantly neurologic phenotype (Fig. 4).

### Clinical characteristics at enrollment in symptomatic patients

The neurologic impairment was more prominent in the V30M (including early and late onset) and non-V30M subgroups as indicated by the derived NIS-LL score (Additional file 3: Table S3). Patients with late-onset V30M had the highest neurologic impairment. Greater cardiac involvement in patients with ATTRwt amyloidosis compared with V30M ATTRv amyloidosis was suggested by greater left ventricular septum thickness and decreased left ventricular ejection fraction (Additional file 3: Table S3). Quality of life, measured by EQ-5D-3L index score and visual analog scale, was comparable between the ATTRwt, V30M, and non-V30M subgroups; however, higher corresponding Norfolk Quality of Life – Diabetic Neuropathy questionnaire scores were observed for the V30M late-onset and non-V30M subgroups.

**Fig. 5:**
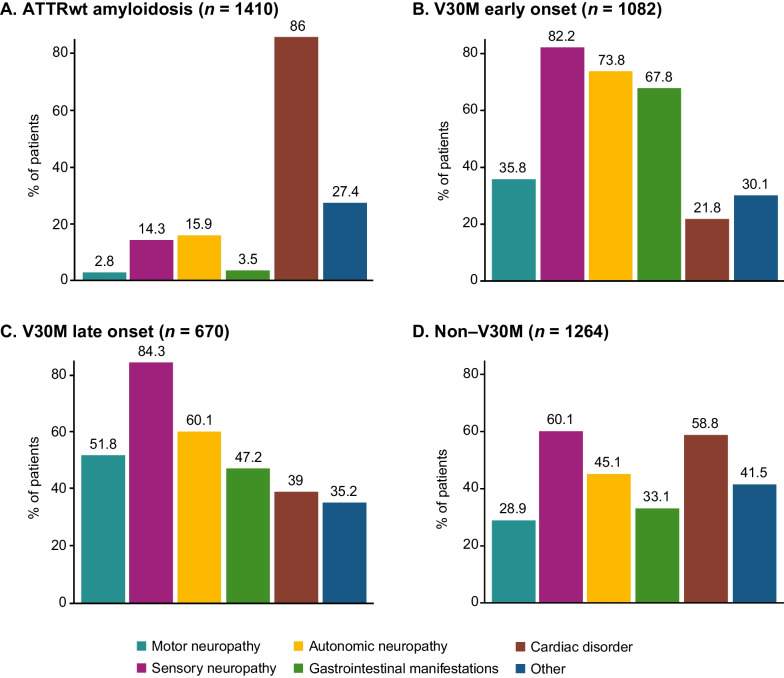
Symptom categories at enrollment in symptomatic patients according to genotype category. V30M early onset and late onset *n* based on all patients with available data for disease diagnosis. ATTRwt amyloidosis = wild-type transthyretin amyloidosis

As observed in last year’s data cutoff [10], sensory neuropathy (54.6%), cardiac disorder (55.4%), and autonomic neuropathy (45.1%) were the most common symptoms corresponding to the symptomatic patients overall. GI manifestations were more common in the V30M (early or late onset) subgroup (59.9%) as compared to the ATTRwt amyloidosis subgroup (3.5%; Fig. 5a, b, c). Similar trends were also observed for autonomic neuropathy and sensory neuropathy with more patients in the V30M (early or late onset) subgroup having these symptom categories. Conversely, cardiac disorders were more frequent among patients with ATTRwt amyloidosis as compared to those with V30M (86.0% vs. 28.4%; Fig. 5a, b, c). Among the non-Val30Met subgroup, sensory neuropathy and cardiac disorders were more frequent (60.1% and 58.8% respectively) as compared with the other symptoms (Fig. 5d).

### Cardiac characteristics at enrollment in symptomatic patients with a predominantly cardiac or mixed phenotype

Heart failure was present in 87.0% of the ATTRwt amyloidosis subgroup as compared to 72.3% of the ATTRv amyloidosis subgroup in the subset of patients with a predominantly cardiac or mixed phenotype (Table 3). A similar greater proportion of patients with abnormal ECG, atrial fibrillation/flutter, pacemaker/implantable cardioverter-defibrillator, and higher values of the cardiac biomarkers (Troponin T, NT-proBNP) were observed in the ATTRwt amyloidosis subgroup as compared to the ATTRv amyloidosis subgroup.

**Table 3:** Cardiac characteristics at enrollment in symptomatic patients with a predominantly cardiac or mixed phenotype

	ATTRwt amyloidosis (*n* = 1410)	ATTRv amyloidosis (*n* = 3017)
Heart failure, *n* (%)	1227 (87.0)	787 (72.3)
NYHA functional class^a^, *n* (%)		
I	126 (10.5)	111 (14.2)
II	729 (60.9)	392 (50.2)
III	315 (26.3)	246 (31.5)
IV	27 (2.3)	32 (4.1)
Abnormal ECG, *n* (%)	1170 (83.0)	670 (61.6)
Atrial fibrillation/flutter, *n* (%)	509 (36.1)	114 (10.5)
Pacemaker implanted, *n* (%)	216 (15.3)	65 (6.0)
ICD implanted, *n* (%)	73 (5.2)	28 (2.6)
NT-proBNP (pg/mL)	*n* = 707	*n* = 255
Median (10th, 90th percentiles)	2146.0 (526.0, 6312.0)	1791.0 (229.0, 6627.0)
Troponin I (ng/mL)	*n* = 112	*n* = 97
Median (10th, 90th percentiles)	0.1 (0.0, 0.3)	0.1 (0.0, 0.5)
Troponin T (ng/mL)	*n* = 270	*n* = 164
Median (10th, 90th percentiles)	0.0 (0.0, 0.1)	0.0 (0.0, 0.2)

*ATTRwt amyloidosis* wild-type transthyretin amyloidosis, *ECG* electrocardiogram, *ICD* implantable cardioverter-defibrillator, *NP-proBNP* N-terminal pro-B-type natriuretic peptide, *NYHA* New York Heart Association, *SD* standard deviation

^a^Denominator for NYHA functional class is the total of non-missing records

### Neurologic characteristics at enrollment in symptomatic patients with a predominantly neurologic or mixed phenotype

Most patients with a predominantly neurologic or mixed phenotype had a score of I (56.9%) or II (19.1%) on the mPND (Additional file 4: Table S4). Patients with V30M late-onset disease had greater walking impairment than those with V30M early-onset disease (mPND > II, 24.7% vs. 9.1%).

## Discussion

This 15-year global overview of > 6000 patients with ATTR amyloidosis and asymptomatic gene carriers is the largest analysis of ATTR amyloidosis to date. The findings presented in this study are largely consistent with the data from the last annual assessment [10].

In this study, V30M and the associated predominantly neurologic phenotype continued to be more prevalent in Europe, South America, and Japan, whereas wild-type disease and the predominantly cardiologic phenotype were most common in North America. Notably, for all genotype subgroups (ATTRwt amyloidosis, early- and late-onset V30M, non-V30M), the highest rate of predominantly cardiologic patients was always found in North America. The reasons for the differences observed are unknown: it has been suggested that they could be related to differences in the age of the populations, the use of scintigraphy imaging techniques in clinical practice, reliance on and/or expertise in the performance and interpretation of endomyocardial biopsy specimens, and specialty of THAOS investigators (e.g., neurologist vs. cardiologist) [12]. However, they might also reflect the true differences in the prevalence of the condition, suggesting a possible role of ambient or socioeconomic factors in increasing the predisposition to cardiac disease. Finally, non-V30M genotypes were highly represented in some individual European, American, and Asian countries (e.g., Mexico, Bulgaria, Denmark, Israel, Italy, Romania, Turkey, Malaysia, and Taiwan).

Male predominance among symptomatic patients was observed in this analysis for all subgroups. However, in the early-onset V30M subgroup, this difference was less pronounced (males, 52.2%; females, 47.8%), consistent with the findings of a Japanese nationwide survey [13]. In contrast, a higher proportion of asymptomatic carriers were female than male, as suggested in previous studies that recorded lower disease penetrance in females, especially those associated with specific genotypes or cardiac manifestation of ATTR amyloidosis [14–18].

As expected, patients with ATTRwt amyloidosis were the oldest at symptom onset (mean age, 72.3 years), which occurred almost 10 years after the late-onset V30M subgroup and > 15 years after the non-V30M subgroup. Patients with early-onset V30M were the youngest at disease onset (mean age, 33.8 years) and received an ATTR amyloidosis diagnosis earlier than the other genotype subgroups (0.9–1.7 years before). This last finding may contribute to the fact that these patients presented with a significant minor grade of walking impairment (9.1% of patients with mPND score ≥ 2) as compared to the other genotypes (20.4% in ATTRwt, 22.7% in late-onset V30M, 18.4% in non-V30M). It should also be noted that, despite everything, patients with early-onset V30M received a diagnosis after a significant amount of time (a mean of 2.8 years after symptom onset). This delay could result from the rarity of ATTR amyloidosis, the wide spectrum of possible symptoms, and the likely presence of confounding comorbidity (especially for patients with ATTRwt amyloidosis).

Compared with last year’s analysis, the proportion of predominantly neurologic patients remained stable (from 40.1 to 38.7%). However, we observed a considerable overall reduction of predominantly cardiac phenotype (from 40.7 to 31.9%) and an increase in mixed phenotype (from 16.6 to 24.5%). This difference was mainly influenced by a change in the definition of the mixed phenotype in THAOS, namely that an mPND score ≥ I is now considered a definitely ATTR amyloidosis–related neurologic symptom, whereas before it was not. As a result of this modification, among the four subgroups of patients (ATTRwt, early- and late-onset V30M, non-V30M), phenotype distribution varied primarily in the ATTRwt subgroup. In these patients, mixed phenotype went from 9.8% as observed in the last year [10] to 23.9%, with a decrease of predominantly cardiac phenotype (from 89 to 76.1%). The global geographic distribution of the mixed phenotype illustrated it was more prevalent in Asia for the ATTRwt and V30M subgroups, and in South America for the non-V30M subgroup.

THAOS has notable strengths in that the registry represents a large, geographically diverse group of countries and study sites worldwide, and provides a more accurate global picture of the current state of ATTR amyloidosis. The large study population of > 6000 patients is a further strength of this analysis. Study limitations include potential differences among study sites in data collection methods, and that the inclusion criteria for THAOS have evolved over time. In addition, temporal bias may have played a role in these findings, as the number and specialties of study sites increased and changed over time, which may have been the greatest influential factor in the patterns observed. Referral bias may have impacted phenotype categorization since cardiac centers may not conduct comprehensive neurologic assessments and vice versa. This could have resulted in an under-representation of the mixed phenotype. In addition, recruitment rates with study sites may vary over time, and registry data, by nature, are not always complete and are limited by the data imputed. Lastly, management of ATTR amyloidosis has evolved over the last few decades and clinical awareness has increased given the emergence of disease-modifying treatments; therefore, patients enrolled in the later years of THAOS likely received more comprehensive assessments than those enrolled in the earlier era.

## Conclusion

This analysis of > 6000 patients and asymptomatic *TTR* gene carriers from THAOS continues to underscore the heterogeneity and increasing awareness of ATTR amyloidosis. The mixed phenotype and multisystemic involvement are increasingly recognized, highlighting the need for a consistent, multidisciplinary approach to the management of ATTR amyloidosis.

### Supplementary Information

**Additional file 1: Table S1** Most frequent genotypes recorded at enrollment in the overall population

**Additional file 2: Table S2** Distribution of phenotype at enrollment in symptomatic patients according to genotype category

**Additional file 3: Table S3** Clinical characteristics and patient-reported outcomes at enrollment in symptomatic patients according to genotype category

**Additional file 4: Table S4** Neurologic characteristics at enrollment in symptomatic patients with a predominantly neurologic or mixed phenotype

## Data Availability

Upon request, and subject to review, Pfizer will provide the data that support the findings of this study. Subject to certain criteria, conditions and exceptions, Pfizer may also provide access to the related individual de-identified participant data. See https://www.pfizer.com/science/clinical-trials/trial-data-and-results for more information.
