# Supplementary material for: A 15-year consolidated overview of data in over 6000 patients from the Transthyretin Amyloidosis Outcomes Survey (THAOS)
Source: Orphanet J Rare Dis. 2023 Nov 10;18:350. doi: 10.1186/s13023-023-02962-5 (PMC10636983; doi:10.1186/s13023-023-02962-5)
Supplement: Supplementary file 1 — Additional file 1: Table S1 Most frequent genotypes recorded at enrollment in the overall population [file 13023_2023_2962_MOESM1_ESM.docx]

**Supplementary Table 1** Most frequent genotypes recorded at
enrollment in the overall population

| **Genotype, *n* (%)** | **Overall**  **(*N* = 6368)** |
| --- | --- |
| V30M (p.V50M)^a^ | 3055 (48.0) |
| Wild-type | 1605 (25.2) |
| V122I (p.V142I) | 381 (6.0) |
| E89Q (p.E109Q) | 156 (2.4) |
| T60A (p.T80A) | 144 (2.3) |
| S50R (p.S70R) | 96 (1.5) |
| S77Y (p.S97Y) | 89 (1.4) |
| I68L (p.I88L) | 81 (1.3) |
| F64L (p.F84L) | 77 (1.2) |
| I107V (p.I127V) | 50 (0.8) |
| D38A (p.D58A) | 48 (0.8) |
| E89K (p.E109K) | 34 (0.5) |
| G47A (p.G67A) | 31 (0.5) |
| V20I (p.V40I) | 29 (0.5) |
| A97S (p.A117S) | 26 (0.4) |
| L111M (p.L131M) | 23 (0.4) |
| V28M (p.V48M) | 21 (0.3) |
| E54Q (p.E74Q) | 20 (0.3) |
| delV122 (p.delV142) | 20 (0.3) |
| S77F (p.S97F) | 18 (0.3) |
| H88R (p.H108R) | 17 (0.3) |
| V122A (p.V142A) | 17 (0.3) |
| T49A (p.T69A) | 15 (0.2) |
| P24S (p.P44S) | 13 (0.2) |
| Y114C (p.Y134C) | 13 (0.2) |
| S52P (p.S72P) | 12 (0.2) |
| S23N (p.S43N) | 10 (0.2) |
| T59K (p.T79K) | 10 (0.2) |

Genotypes recorded in ≥ 10 patients at enrollment are shown

^a^ Includes 97 patients with G6S (p.G26S)/V30M (p.V50M) mutations
