# Supplementary material for: A 15-year consolidated overview of data in over 6000 patients from the Transthyretin Amyloidosis Outcomes Survey (THAOS)
Source: Orphanet J Rare Dis. 2023 Nov 10;18:350. doi: 10.1186/s13023-023-02962-5 (PMC10636983; doi:10.1186/s13023-023-02962-5)
Supplement: Supplementary file 2 — Additional file 2: Table S2 Distribution of phenotype at enrollment in symptomatic patients according to genotype category [file 13023_2023_2962_MOESM2_ESM.docx]

**Supplementary Table 2** Distribution of phenotype at enrollment in symptomatic patients according to
genotype category

| **Phenotype category, *n* (%)** | **Overall**  **(*n* = 4428)** | **ATTRwt amyloidosis**  **(*n* = 1410)** | V30M overall (***n*** = 1753) | V30M early onset  (***n*** = 1082) | V30M late onset  (***n*** = 670) | Non-V30M  (***n*** = 1264) |
| --- | --- | --- | --- | --- | --- | --- |
| All symptomatic patients |  |  |  |  |  |  |
| Predominantly cardiac | 1413 (31.9) | 1073 (76.1) | 45 (2.6) | 15 (1.4) | 29 (4.3) | 295 (23.3) |
| Predominantly neurologic | 1712 (38.7) | 0 | 1254 (71.5) | 855 (79.0) | 399 (59.6) | 457 (36.2) |
| Mixed | 1085 (24.5) | 337 (23.9) | 354 (20.2) | 149 (13.8) | 205 (30.6) | 394 (31.2) |
| No phenotype | 218 (4.9) | 0 | 100 (5.7) | 63 (5.8) | 37 (5.5) | 118 (9.3) |
| North America, *n* | 1352 | 805 | 36 | 5 | 31 | 511 |
| Predominantly cardiac | 864 (63.9) | 680 (84.5) | 8 (22.2) | 1 (20.0) | 7 (22.6) | 176 (34.4) |
| Predominantly neurologic | 174 (12.9) | 0 | 17 (47.2) | 4 (80.0) | 13 (41.9) | 157 (30.7) |
| Mixed | 253 (18.7) | 125 (15.5) | 8 (22.2) | 0 | 8 (25.8) | 120 (23.5) |
| No phenotype | 61 (4.5) | 0 | 3 (8.3) | 0 | 3 (9.7) | 58 (11.4) |
| South America, *n* | 259 | 11 | 206 | 143 | 62 | 42 |
| Predominantly cardiac | 19 (7.3) | 7 (63.6) | 2 (1.0) | 0 | 1 (1.6) | 10 (23.8) |
| Predominantly neurologic | 170 (65.6) | 0 | 158 (76.7) | 118 (82.5) | 40 (64.5) | 12 (28.6) |
| Mixed | 65 (25.1) | 4 (36.4) | 42 (20.4) | 23 (16.1) | 19 (30.6) | 19 (45.2) |
| No phenotype | 5 (1.9) | 0 | 4 (1.9) | 2 (1.4) | 2 (3.2) | 1 (2.4) |
| Europe, *n* | 2570 | 573 | 1393 | 871 | 522 | 603 |
| Predominantly cardiac | 492 (19.1) | 375 (65.4) | 29 (2.1) | 12 (1.4) | 17 (3.3) | 88 (14.6) |
| Predominantly neurologic | 1252 (48.7) | 0 | 1006 (72.2) | 688 (79.0) | 318 (60.9) | 245 (40.6) |
| Mixed | 681 (26.5) | 198 (34.6) | 268 (19.2) | 112 (12.9) | 156 (29.9) | 215 (35.7) |
| No phenotype | 145 (5.6) | 0 | 90 (6.5) | 59 (6.8) | 31 (5.9) | 55 (9.1) |
| Japan, *n* | 151 | 7 | 114 | 62 | 52 | 30 |
| Predominantly cardiac | 18 (11.9) | 5 (71.4) | 6 (5.3) | 2 (3.2) | 4 (7.7) | 7 (23.3) |
| Predominantly neurologic | 84 (55.6) | 0 | 71 (62.3) | 45 (72.6) | 26 (50.0) | 13 (43.3) |
| Mixed | 47 (31.1) | 2 (28.6) | 35 (30.7) | 14 (22.6) | 21 (40.4) | 10 (33.3) |
| No phenotype | 2 (1.3) | 0 | 2 (1.8) | 1 (1.6) | 1 (1.9) | 0 |
| Other Asia, *n* | 96 | 14 | 4 | 1 | 3 | 78 |
| Predominantly cardiac | 20 (20.8) | 6 (42.9) | 0 | 0 | 0 | 14 (17.9) |
| Predominantly neurologic | 32 (33.3) | 0 | 2 (50.0) | 0 | 2 (66.7) | 30 (38.5) |
| Mixed | 39 (40.6) | 8 (57.1) | 1 (25.0) | 0 | 1 (33.3) | 30 (38.5) |
| No phenotype | 5 (5.2) | 0 | 1 (25.0) | 1 (100.0) | 0 | 4 (5.1) |

V30M early onset and late onset *n* based on all patients with available data for disease diagnosis; patients with unknown phenotype
were symptomatic patients who did not fulfill criteria for any of the other phenotype categories

ATTRwt amyloidosis = wild-type transthyretin amyloidosis
