# Supplementary material for: A 15-year consolidated overview of data in over 6000 patients from the Transthyretin Amyloidosis Outcomes Survey (THAOS)
Source: Orphanet J Rare Dis. 2023 Nov 10;18:350. doi: 10.1186/s13023-023-02962-5 (PMC10636983; doi:10.1186/s13023-023-02962-5)
Supplement: Supplementary file 3 — Additional file 3: Table S3 Clinical characteristics and patient-reported outcomes at enrollment in symptomatic patients according to genotype category [file 13023_2023_2962_MOESM3_ESM.docx]

**Supplementary Table 3** Clinical characteristics and patient-reported outcomes at enrollment in symptomatic patients according to genotype category

|  | **Overall**  **(*n* = 4428)** | **ATTRwt amyloidosis**  **(*n* = 1410)** | V30M early onset  (***n*** = 1082) | V30M late onset  (***n*** = 670) | Non-V30M  (***n*** = 1264) |
| --- | --- | --- | --- | --- | --- |
| BMI (kg/m^2^), *n* | 4304 | 1383 | 1058 | 644 | 1217 |
| Mean (SD) | 25.9 (14.62) | 28.1 (21.39) | 23.3 (4.89) | 25.7 (11.77) | 25.9 (11.42) |
| Modified BMI^a^ (g/L), *n* | 2910 | 896 | 841 | 473 | 700 |
| Mean (SD) | 1038.1 (231.77) | 1072.9 (202.27) | 1007.4 (239.88) | 1033.2 (228.00) | 1033.8 (253.37) |
| Sitting systolic BP, *n* | 4297 | 1386 | 1053 | 639 | 1217 |
| Mean (SD) | 122.5 (18.86) | 121.4 (18.06) | 123.2 (15.75) | 133.2 (20.36) | 117.5 (19.11) |
| Sitting diastolic BP, *n* | 4295 | 1386 | 1053 | 638 | 1216 |
| Mean (SD) | 75.1 (11.84) | 72.9 (11.54) | 78.1 (11.49) | 77.8 (11.13) | 73.7 (12.06) |
| Derived NIS-LL score, n | 1761 | 167 | 788 | 314 | 492 |
| Mean (SD) | 16.5 (20.64) | 4.3 (6.02) | 16.5 (20.38) | 26.1 (24.04) | 14.4 (19.15) |
| Reflex score, *n* | 2699 | 339 | 996 | 518 | 846 |
| Mean (SD) | 7.9 (3.20) | 9.3 (1.76) | 8.5 (2.50) | 6.3 (3.72) | 7.5 (3.55) |
| Motor score, *n* | 2581 | 457 | 942 | 425 | 757 |
| Mean (SD) | 151.8 (19.24) | 158.2 (13.34) | 151.3 (19.52) | 145.9 (22.27) | 151.9 (18.96) |
| Sensory score, *n* | 1527 | 123 | 710 | 272 | 422 |
| Mean (SD) | 103.9 (25.89) | 121.8 (6.12) | 100.6 (26.48) | 93.1 (30.82) | 111.2 (19.63) |
| LV septum thickness (mm), *n* | 2342 | 1074 | 239 | 274 | 754 |
| Mean (SD) | 15.8 (5.18) | 17.1 (3.59) | 10.3 (2.35) | 14.8 (4.43) | 15.9 (6.54) |
| LV ejection fraction (%), *n* | 2217 | 1085 | 195 | 192 | 744 |
| Mean (SD) | 52.4 (13.64) | 48.5 (12.26) | 62.7 (10.77) | 61.5 (9.38) | 52.9 (14.54) |
| EQ-5D-3L: VAS overall health, *n* | 2783 | 873 | 824 | 395 | 690 |
| Mean (SD) | 66.1 (20.47) | 66.1 (19.40) | 69.8 (18.87) | 60.9 (21.84) | 64.7 (22.01) |
| EQ-5D-3L: Derived index, *n* | 2831 | 888 | 830 | 404 | 708 |
| Mean (SD) | 0.75 (0.202) | 0.80 (0.165) | 0.75 (0.192) | 0.67 (0.233) | 0.73 (0.217) |
| Norfolk Total QoL-DN score | 2831 | 875 | 834 | 411 | 710 |
| Mean (SD) | 31.6 (28.79) | 23.2 (20.69) | 29.9 (27.54) | 45.3 (33.22) | 35.8 (32.06) |
| Karnofsky Performance Status^b^, *n* (%) |  |  |  |  |  |
| 10–30 | 12 (0.4) | 3 (0.5) | 1 (0.1) | 0 | 8 (1.0) |
| 40–60 | 458 (15.4) | 98 (16.0) | 91 (9.5) | 122 (22.5) | 147 (16.9) |
| 70–90 | 2153 (72.2) | 459 (75.0) | 745 (77.7) | 376 (69.4) | 572 (65.9) |
| 100 | 359 (12.0) | 52 (8.5) | 122 (12.7) | 44 (8.1) | 141 (16.2) |

V30M early onset and late onset *n* based on all patients with available data for disease diagnosis

^a^ Calculated by multiplying BMI by serum albumin levels to compensate for fluid accumulation

^b^ Karnofsky Performance Status percentages are based on non-missing observations

ATTR amyloidosis = transthyretin amyloidosis; ATTRwt amyloidosis = wild-type transthyretin amyloidosis; BMI = body mass index; BP = blood pressure; LV = left ventricular; mPND = modified Polyneuropathy Disability; NIS-LL = Neuropathy Impairment Score in the Lower Limbs; Norfolk Total QoL-DN = Norfolk Quality of Life – Diabetic Neuropathy questionnaires; VAS = visual analog scale
