# Supplementary material for: A 15-year consolidated overview of data in over 6000 patients from the Transthyretin Amyloidosis Outcomes Survey (THAOS)
Source: Orphanet J Rare Dis. 2023 Nov 10;18:350. doi: 10.1186/s13023-023-02962-5 (PMC10636983; doi:10.1186/s13023-023-02962-5)
Supplement: Supplementary file 4 — Additional file 4: Table S4 Neurologic characteristics at enrollment in symptomatic patients with a predominantly neurologic or mixed phenotype [file 13023_2023_2962_MOESM4_ESM.docx]

**Supplementary Table 4** Neurologic characteristics at enrollment in symptomatic patients with a predominantly neurologic or mixed phenotype

|  | **Overall**  **(*n* = 4428)** | **ATTRwt amyloidosis**  **(*n* = 1410)** | V30M early onset  (***n*** = 1082) | V30M late onset  (***n*** = 670) | Non-V30M  (***n*** = 1264) |
| --- | --- | --- | --- | --- | --- |
| Patients with data available, *n* | 2369 | 256 | 942 | 523 | 647 |
| mPND score at enrollment, *n* (%) |  |  |  |  |  |
| 0 | 183 (7.7) | 28 (10.9) | 57 (6.1) | 17 (3.3) | 80 (12.4) |
| I | 1348 (56.9) | 135 (52.7) | 628 (66.7) | 242 (46.3) | 343 (53.0) |
| II | 452 (19.1) | 41 (16.0) | 171 (18.2) | 135 (25.8) | 105 (16.2) |
| IIIa | 193 (8.1) | 36 (14.1) | 47 (5.0) | 62 (11.9) | 48 (7.4) |
| IIIb | 119 (5.0) | 11 (4.3) | 24 (2.5) | 37 (7.1) | 47 (7.3) |
| IV | 74 (3.1) | 5 (2.0) | 15 (1.6) | 30 (5.7) | 24 (3.7) |

V30M early onset and late onset *n* based on all patients with available data for disease diagnosis

ATTRwt amyloidosis = wild-type transthyretin amyloidosis; mPND = modified Polyneuropathy Disability
